# Synergistic Effect of Al_2_O_3_ Inclusion and Pearlite on the Localized Corrosion Evolution Process of Carbon Steel in Marine Environment

**DOI:** 10.3390/ma11112277

**Published:** 2018-11-14

**Authors:** Chao Liu, Xuequn Cheng, Zeyu Dai, Ryan Liu, Ziyu Li, Liying Cui, Mindong Chen, Le Ke

**Affiliations:** 1 Institute of Advanced Materials & Technology, University of Science and Technology Beijing, Beijing 100083, China; liuchaoustb@163.com (C.L.); daizeyuustb@163.com (Z.D.); zyli16@163.com (Z.L.); mandytsui@163.com (L.C.); euacfer@163.com (M.C.); ustb_kl@163.com (L.K.); 2 The Calverton School, Huntingtown, MD 20639, USA; wliu00@calvertonschool.org

**Keywords:** carbon steel, localized corrosion, inclusion, pearlite

## Abstract

The initiation and evolution of the localized corrosion in carbon steel were investigated in a simulated marine environment of Xisha Island in the South China Sea. In the initial stage, localized corrosion occurred in the form of corrosion spot. The localized corrosion morphology and electrochemical information during corrosion process were tracked by field emission scanning electron microscopy energy dispersive spectrometry (FE-SEM-EDS), scanning vibrating electrode technique (SVET) and scanning Kelvin probe force microscopy (SKPFM). Localized corrosion was induced by the microcrevices around Al_2_O_3_ inclusions. The occluded cells and oxygen concentration cell formed in the pits could accelerate the localized corrosion. Pearlite accelerated the dissolution of the inside and surrounding ferrite via the galvanic effect between Fe_3_C and ferrite. Overall, the localized corrosion was initiated and evaluated under a synergistic effect of crevice corrosion, occluded cells, oxygen concentration cell and the galvanic couple between FeC_3_ and ferrite.

## 1. Introduction

High strength steels have been used globally for the construction of road installations, electricity posts, utility towers, guide rails, ornamental sculptures and facades and oil and gas pipelines in the marine environment. During the service processes of steel, localized corrosion is one of the most serious problems, which causes catastrophic failure of metallic structures, especially in the harsh marine environment [1,2,3,4].

As a typical form of localized corrosion, pitting has been described as a random, sporadic and stochastic process, making it extremely difficult to predict and control [5]. Pitting has long been explained as a result of the variation of electrochemical homogeneity in materials, such as the appearance of inclusion [2,6,7,8]. In the initial stage of pitting process, some corrosion spots are formed in the localized corrosion areas on metal surface due to the dissolution of nanoscale secondary ion sites in the stainless steel, such as MnS [9]. A similar phenomenon has also been observed in ultra-low-carbon bainitic steel [10]. A galvanic couple is generally considered to exist between the steel matrix and the MnS inclusions, which is a common inclusion type [10,11]. In carbon steel, MnS can accelerate the dissolution of the matrix as a cathodic phase [10]. In carbon steel, cementite (Fe_3_C) is cathodic to ferrite [12], thus pitting is observed in the adjacent ferrite [13]. The widespread distribution of Fe_3_C might result in multiple localized corrosion with limited depth, which is regarded as a kind of localized general corrosion [14]. It has been reported that there is an interactive effect between cementite and MnS inclusion on corrosion. Both MnS and cementite are cathodic phase compared to the matrix, and localized corrosion area can form around them as an anodic area. Meanwhile, for each anodic area, there is an associated cathodic area (and current) that supports the anodic reaction [15]. The interactive effect between cementite and MnS is reported to be closely related to the ratio of carbon to sulfur content (C/S) in the steel [11]. Deeper pitting possesses lower C/S ratio in carbon steel.

With the development of desulfurization technology during smelting process, the content of sulfur decreased significantly. Hence, MnS inclusion with smaller amount and size is negligible for corrosion effect. Al_2_O_3_ inclusion is the most common inclusion in Al-killed steel. We preciously clarified the mechanism of pitting initiation process by Al_2_O_3_ inclusion [16]. However, the synergistic effect of Al_2_O_3_ inclusion and pearlite on the localized corrosion evolution process of carbon steel remains unclear. Therefore, we thoroughly explored the synergistic effect of Al_2_O_3_ inclusion and pearlite on the localized corrosion evolution process of Q460NH steel under a simulative marine environment. Scanning vibrating electrode technique (SVET) and scanning Kelvin probe force microscopy (SKPFM) were used to investigate the localized electrochemical information during corrosion. Field emission–-scanning electron microscopy–energy dispersive spectrometry (FE-SEM-EDS) was employed to characterize the microstructures.

## 2. Experimental

### 2.1. Specimen Preparation

All specimens were made from a carbon steel with a composition (wt %) of 0.03 C, 0.25 Si, 0.1 Mn, 0.011 P, 0.002 S, 0.4 Cu, 1.2 Cr, 0.3 Ni, 0.024 Al, 0.08 Nb and Fe for balance. The specimen with a size of 10 mm × 10 mm × 6 mm was mechanically ground with silicon–carbide papers down to 4000 grit and then polished with a 0.5 μm diamond. The specimens were then ultrasonically rinsed in ethanol. Prior to the microstructure observation, the specimens were mechanically polished and then etched by 4% nital solution. The microstructures of the specimen before and after etching were observed with a JEOL JSM-7100F Field emission scanning electron microscopy (FE-SEM) before and after etching the specimens. The elemental distributions of the inclusions were identified by the energy dispersive spectrometer (EDS). An accelerating voltage of 30 kV, a probe current of 10 nA, and a working distance of 10 mm were fixed for both secondary electrons images and EDS analysis.

### 2.2. In-Situ Micro-Electrochemical Measurements

SKPFM was used to investigate the electrochemical nature of the inclusions in Q460NH steel; in particular, the Volta potential was investigated with respect to the matrix. The SKPFM measurements were conducted at room temperature in air using a commercial atomic force microscope (Park Systems XE-100). Cr/Au-coated tips on conductive cantilevers (NSC36/Cr-Au) with a nominal resonant frequency of about 65 kHz and a nominal spring constant of about 0.6 N/m were used. The thickness of coating on both sides was ~20 nm, and the radius of curvature was ~50 nm. A single-pass methodology was employed, in which the topography and corresponding contact potential difference between the probe and the sample were simultaneously measured. The samples were scanned at a rate of 0.1 Hz. The contact potential signals were inverted to reflect the Volta potential of the surface.

SVET (Scanning Vibrating Electrode Technique) measures the local ionic current by scanning about 100 µm above a sample surface with a vibrating probe. It picks up small potential variations between the probe and a reference electrode at certain frequencies with a lock-in amplifier filtering out electrical noise and subsequently converts the potential difference into an ionic current density by using Ohm’s law. The SVET instrument was manufactured by Applicable Electronics, LLC (Sandwich, MA, USA) and controlled by the ASET 2.10 program developed by Science Wares, Inc (Sandwich, MA, USA). A 20 μm diameter platinum black sphere was electrodeposited on the tip. The microelectrode vibrates in two directions, one parallel (*y* axis) and the other normal (*z* axis) to the sample surface, sensing the electric field in the two directions; however, in corrosion, the signals for the *x* vibration are seldom used. Further details can be found in the literature [17,18]. In the SVET test, an about 1–4 mm^2^ area was selected to track the micro-electrochemical signal. To ensure the conveniences of measurement, the sample surface was covered with paraffin to isolate unmeasured face and solutions.

### 2.3. Characterization of Corrosion Morphology

Immersion test is an effective method to observe the corrosion morphology on the material surface [19,20,21]. An immersion solution consisting of 0.1 wt % NaCl, 0.05 wt % Na_2_SO_4_ and 0.05 wt % CaCl_2_ was employed as a simulated solution of the thin electrolyte film that is known to form in the humid atmosphere on Xisha Island in the South China Sea [22]. The corrosion morphology was observed by FE-SEM before and after removing the corrosion products with a solution containing HCl and hexamethylenetetramine [23]. Thereafter, the specimen was washed with deionized water and alcohol and then blow dried.

## 3. Results

### 3.1. Inclusions and Microstructure Characterization

**Figure 1:**
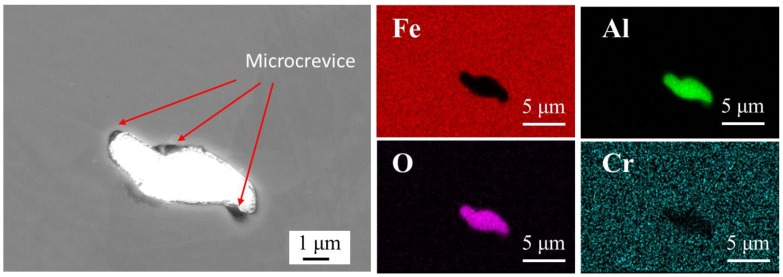
SEM image and EDS maps of inclusions in the steel.

Inclusions in the steel were analyzed by FE-SEM-EDS prior to etching. As shown in Figure 1, Al_2_O_3_ inclusions existed in the steel. This result is consistent with the SEM-EDS observations in Al-killed alloyed steel [24]. The size of Al_2_O_3_ inclusions ranged from 2 to 7 μm. Some microcrevices were observed at the matrix–inclusion interfaces (Figure 1), which was mainly due to the differences of the strain values [25,26] and coefficients of thermal expansion [27]. Similar microcrevices have been observed in previous investigations [25,28,29,30].

**Figure 2:**
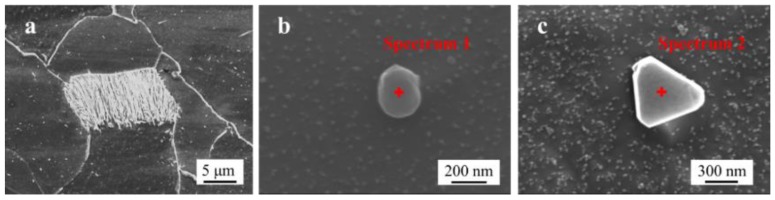
SEM images of different microstructures in the steel: (**a**) pearlite in the steel; and (**b**,**c**) nanoscale inclusions in the steel.

After analyzing the inclusions in the steel, the sample was etched with a 4% Nital solution. As shown in Figure 2, the microstructures in the steel were pearlite and ferrite (Figure 2a). Two different types of nanoscale inclusions were observed in the steel: MnS (Figure 2b) and Al_2_O_3_–MnS (Figure 2c). Their composition is shown in Table 1, and the size of these nanoscale inclusions ranged 100–500 nm.

### 3.2. Micro-Electrochemical Property Differences

**Figure 3:**
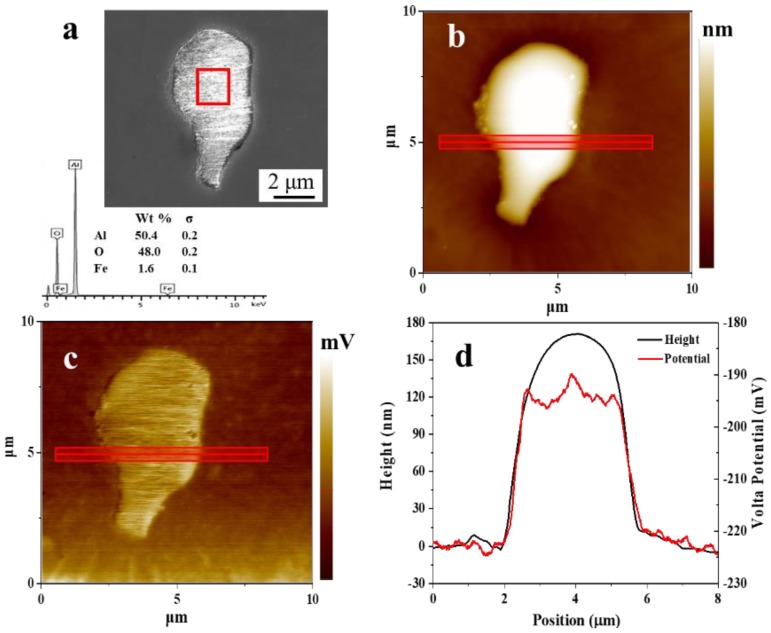
SEM image, AFM topography map and SKPFM Volta potential map of an Al_2_O_3_ inclusion in the surface of the experimental steel: (**a**) SEM image of the Al_2_O_3_ inclusion; (**b**) topographical map (color bar: 250 nm range); (**c**) Volta potential map (color bar: 70 mV range); and (**d**) results of the line-scan shown in (**b**,**c**).

SKPFM measurements were conducted to obtain abundant information regarding the electrochemical characteristics associated with the local surface inhomogeneities. Figure 3 shows the FE-SEM image and EDS result (Figure 3a) accompanied by the AFM topography map (Figure 3b), and SKPFM Volta potential map (Figure 3c) of an Al_2_O_3_ inclusion. A line profile analysis result of the AFM/SKPFM images is shown in Figure 3d.

As shown in Figure 3d, the Al_2_O_3_ inclusions exhibited a higher potential than the matrix, which proved that the inclusion was more stable than the matrix, and corrosion was very likely to initiate from the matrix. After measuring many areas on the surface of the experimental steel (about 25), the Volta potential of the Al_2_O_3_ inclusions was measured to be 30 ± 8 mV higher than that of the matrix. The potential differences between the inclusions and matrix fluctuated slightly along with the size of the inclusions: smaller inclusions led to a smaller difference of the Volta potential value between the matrix and inclusions [4,31,32]. This might be because the Volta potential of a small surface feature measured by SKPFM includes a contribution from the surrounding matrix and was thus an average value over the surrounding region [33,34].

### 3.3. Evolution Tracking of the Localized Corrosion

**Figure 4:**
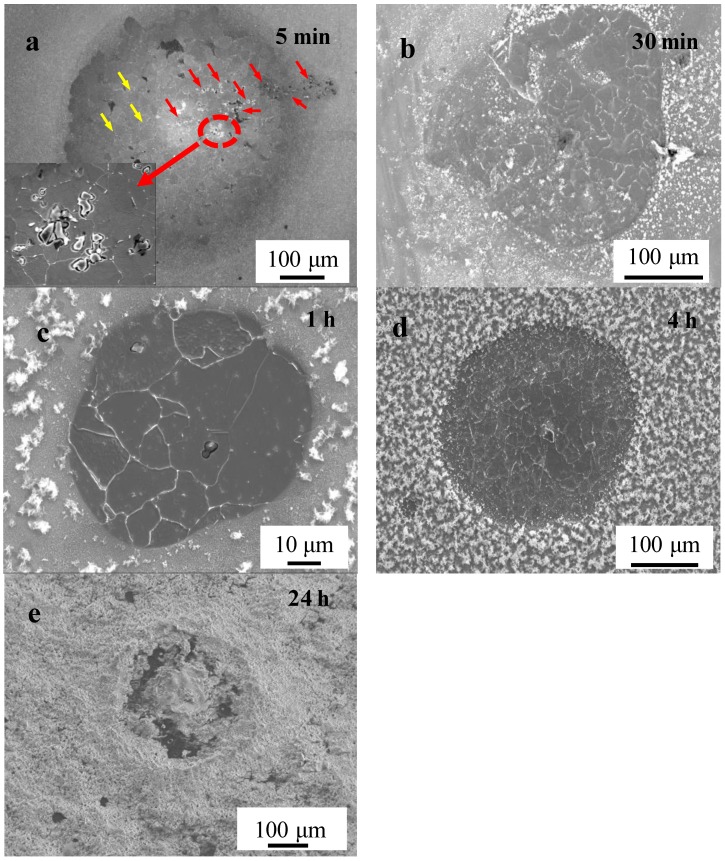
The morphology of localized corrosion on specimens after immersion test: (**a**) 5 min; (**b**) 30 min; (**c**) 1 h; (**d**) 4 h; and (**e**) 24 h.

The evolution process of localized corrosion is shown in Figure 4. The morphology of localized corrosion on specimens was observed after immersing in a simulated marine environment solution for 5 min, 30 min, 1 h, 4 h and 24 h. After immersion for 5 min, localized corrosion was clearly observed on the surface as a corrosion spot (Figure 4a). Many pits around inclusion were observed (marked as red arrows). Simultaneously, intermittent grain boundaries (marked as yellow arrows) could be observed in the corrosion spot result from the slight dissolve of the grain. After various immersion times (Figure 4b–d), similar corrosion spots could be seen on the specimen surface, with different rust amounts and morphologies. As the immersion time increased, the rust layer gradually became denser. After 24 h immersion (Figure 4e), rust completely covered the corrosion spots.

### 3.4. In Suit Tracking of the Microelectrochemical Corrosion Information

To investigate the local electrochemical behavior in the initiation and developing process of the localized corrosion area, the corrosion current was tested. Specimens were corroded spontaneously with a clear separation between the cathode and the anode [35]. During the test, the anodic and cathodic activities were usually maintained at the same locations for several hours, although the location tended to change as the corrosion products began to cover the surface.

**Figure 5:**
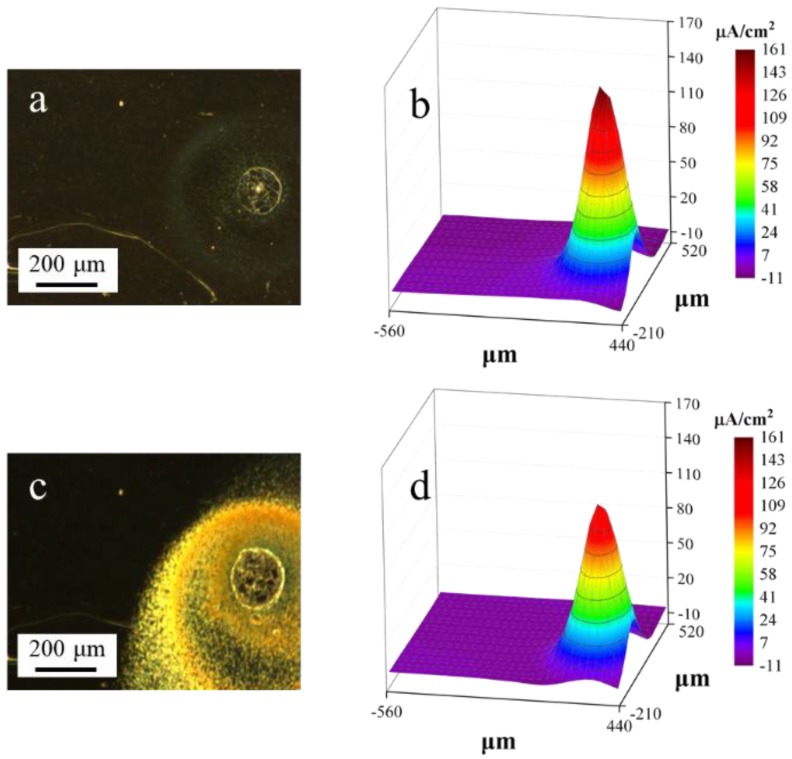
Optical-microscope images (**left**) and ionic current maps (**right**) of the experimental steel during immersion in the solution for: (**a**,**b**) 5 min; and (**c**,**d**) 30 min. Test area size: ~1 mm^2^.

Figure 5 shows the in situ optical images and the corresponding ionic current maps obtained from the SVET measurements. As shown in the optical images in Figure 5a, a clear corrosion spot was formed due to the localized corrosion after being immersed for 5 min. In the corrosion spot, a high anodic corrosion current was detected in the corresponding current map (Figure 5b). The current density peak in the corrosion spot was about 160 μA/cm^2^, with the pre-corroded area as the anode and the originally uncorroded electrode as the cathode. As shown in Figure 5a, a light-yellow circular band area was observed around the corrosion spot. This area resulted from the drifting of the corrosion product from the center of the spot to the outside. Figure 5c shows the morphology of the corrosion spot after 30 min immersion. The shape of the corrosion spot in Figure 5c was similar to that in Figure 5a. The surfaces of both the pre-corroded and the uncorroded areas around the corrosion spot were covered with corrosion products. The current density peak in the center of the corrosion spot was 129 μA/cm^2^ (Figure 5d).

**Figure 6:**
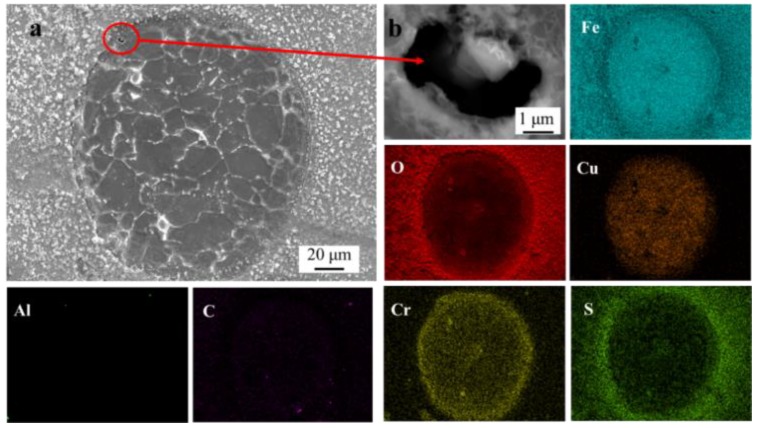
SEM image and EDS maps of the corrosion spot. (**a**) The SEM image of the corrosion spot. (**b**) Pit in the corrosion spot marked in (**a**).

After the SVET test, the corrosion product on the sample was observed by FE-SEM-EDS. Figure 6 shows the SEM image and the corresponding EDS maps of the corrosion spot. An inclusion was present in the corrosion spot, with the corrosion product covering the pit (Figure 6b). This led to the formation of occluded cells with the pits acting as anodes. The EDS maps demonstrated that Fe, O, and S distributed on the entire surface. Moreover, Cu and Cr were found to be enriched on the surface inside the corrosion spot.

**Figure 7:**
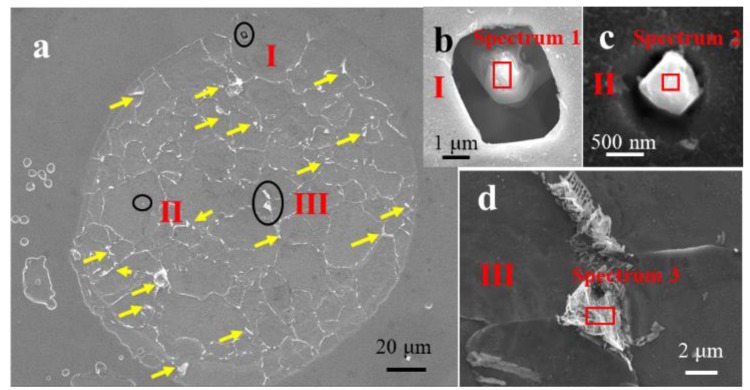
(**a**) SEM image of the corrosion spot after removing the corrosion product; (**b**) inclusion in position I; (**c**) nanoscale inclusion of position II; and (**d**) pearlite in position III.

The corrosion spot was clearly observed after removing the corrosion product (Figure 7a). High-magnification images of the areas marked as I, II and III are presented in Figure 7b–d, respectively. A deep cave pit induced by an Al_2_O_3_ inclusion (with its composition shown in Table 2) is shown in Figure 7b. Another Al_2_O_3_ nanoscale inclusion and open pits were observed in the corrosion spot (Figure 7c). The composition of the nanoscale inclusion is presented in Table 2. No MnS inclusion was observed in the corrosion spot after the corrosion product was removed. These nanoscale inclusions might detach after ultrasonic treatment. No obvious deep pit was observed around the nanoscale inclusion. Figure 7d shows that the pearlite in the steel acted as a flake skeleton in the corrosion spot. Pearlite was made up of lamellar Fe_3_C and ferrite, and lamellar Fe_3_C can accelerate the dissolution of ferrite as a cathodic phase during corrosion process [36,37,38]. Numerous remaining lamellar Fe_3_C (marked with yellow arrows) were observed in the corrosion spot.

**Figure 8:**
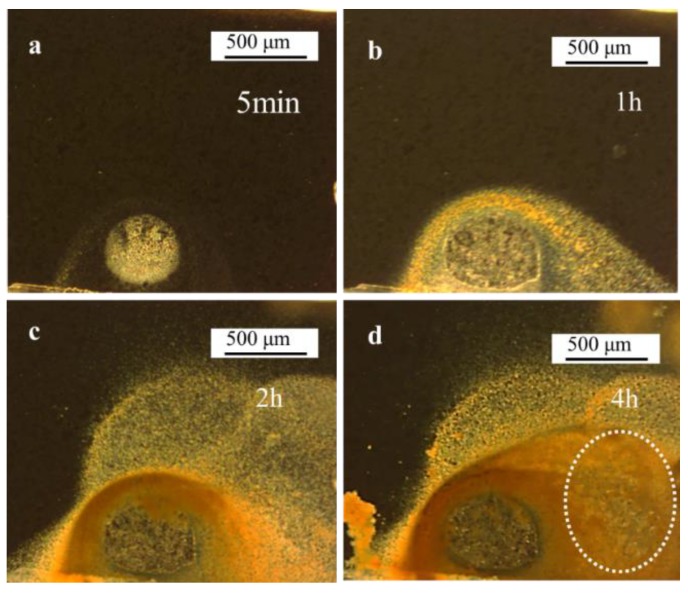
Optical-microscope images (left) of the experimental steel during immersion in the solution in the 4 h SVET test: (**a**) 5 min; (**b**) 1 h; (**c**) 2 h; and (**d**) 4 h.

A 4-h SVET test was conducted to track the propagation of the corrosion spot. As shown in Figure 8a, after immersed in solution for 5 min, a clear corrosion spot covered with rust was observed on the sample surface. After 1 h, the corrosion spot grew larger, and the rust diffused to adjacent areas around the spot (Figure 8b). As the immersion time increased to 2 h, area covered with rust increased obviously (Figure 8c). The color of the rust covered around the boundary of the corrosion spot was deeper than relatively remote areas (Figure 8c), indicating the rust in the deep color area was much thicker. After being immersed for 4 h, the deep color rust covering area extended much further than previously (Figure 8d). In the area marked with a white circle (Figure 8d), some smaller corrosion spots could be observed with thinner rust covering them. The surface of the corrosion spot bottom was rugged. In the early stage of corrosion process, localized corrosion occurred in the smaller test area, and the adjacent steel around the corrosion spot was uncorroded (Figure 8a). With the floating diffusion of the rust generated from the corrosion area, the adjacent area started to be covered by the rust. Subsequently, corrosion initiated at these areas.

**Figure 9:**
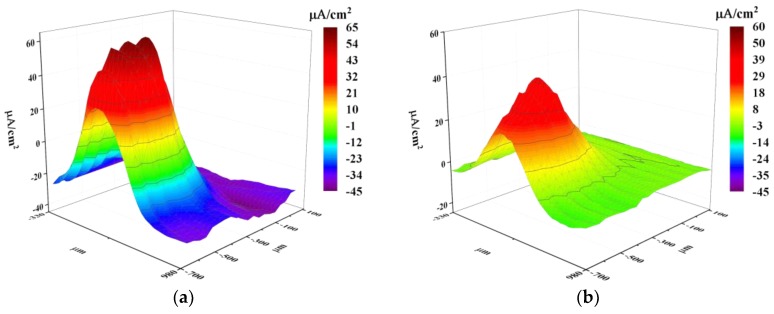

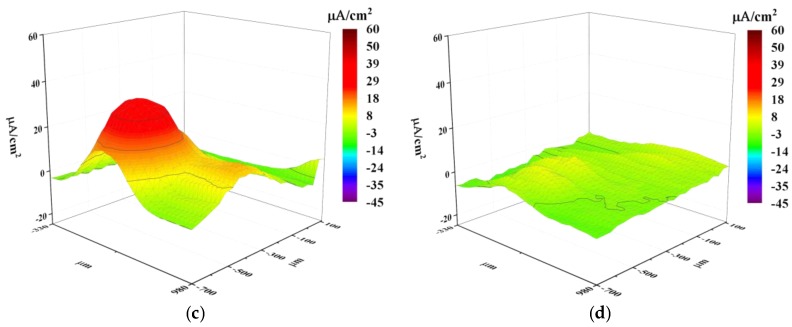
Ionic current maps of the experimental steel during immersion in the solution during the 4-h SVET test: (**a**) 5 min; (**b**) 1 h; (**c**) 2 h; and (**d**) 4 h. Test area size: ~1 mm^2^.

Figure 9 shows the ionic current change during the 4-h SVET test. At 5 min (Figure 9a), the corrosion current was the highest. There was a significant current difference between the corrosion spot and the adjacent area (Figure 9a). The peak current in the corrosion spot decreased over time (Figure 9b–d), and the current difference decreased simultaneously. As shown in Figure 9c, anodic current covering area increased compared with Figure 9a. At 4 h, the anode current decreased to the minimum, while the current of anode corrosion covering area peaked. This can be explained by the shield effect of the rust covered on the surface.

**Figure 10:**
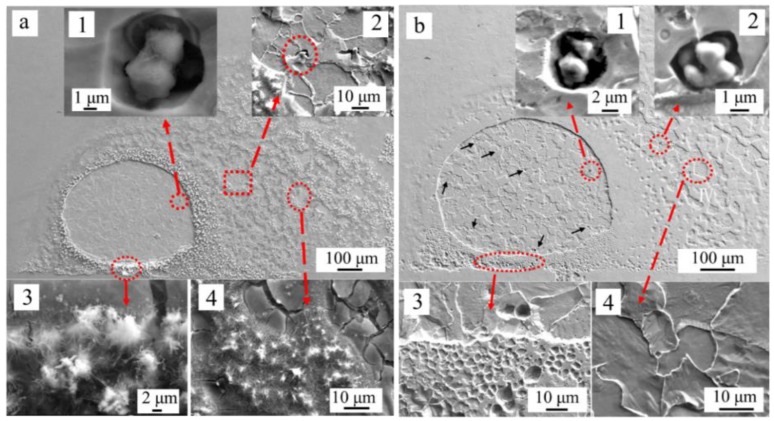
(**a**) SEM image of the corrosion spot before removing the corrosion product, (**b**) SEM image of the corrosion spot after removing the corrosion product.

The morphologies of the corrosion area before and after removing rust are shown in Figure 10. As shown in Figure 10a, a large amount of rust covered around the corrosion spot. Some pits around inclusions were observed inside the corrosion spot (Figure 10(a_1_)), and some pits initiated around inclusions were also observed in the rust covered area slightly away from the corrosion spot (Figure 10(a_2_)). Some needle-like and cotton-like rust was formed at the boundary (Figure 10(a_3_)) of the corrosion spot or in the adjacent area (Figure 10(a_4_)). After the rust was removed, a clear corrosion spot was observed on the sample surface. Some pits initiated around inclusions was observed inside the corrosion spot (Figure 10(b_1_)) and in the adjacent area from the corrosion spot (Figure 10(b_2_)). Moreover, some smaller pits marked with black arrows could be observed inside the corrosion spot (Figure 10b). Figure 10(b_3_,b_4_) show the matrix in the rust covered area was also dissolved. As shown in Figure 10(b_4_), the grain was dissolved obviously. This indicated that corrosion form was transformed to uniform corrosion from localized corrosion after 4 h immersion.

### 3.5. Effect of Inclusion and Pearlite on the Localized Corrosion

As mentioned before, inclusions played an important role in the corrosion process. Deep pits were initiated around them due to the presence of microcrevices (Figure 1) and unstable area (Figure 3).

**Figure 11:**
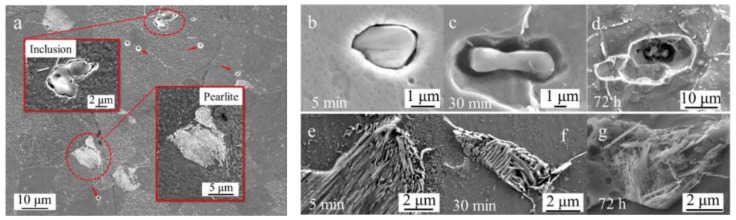
The morphology of samples after a series of immersion test: (**a**,**b**,**e**) 5 min; (**c**,**f**) 30 min; and (**d**,**g**) 72 h.

A series of immersion tests were conducted to trace the initiation and evolution process of pits around inclusions. Figure 11 shows the surface morphology of the sample after a series of immersion tests.

As shown in Figure 11a, after 5 min immersion in the simulated solution of marine environment, the matrix around the inclusions dissolved slightly. The morphology of pits initiated around inclusions differs from the microcrevices occasionally observed at some places of the inclusion/matrix interface. In the early stage of the corrosion process, no significant dissolution of the pearlite occurred after being immersed for 5 min. Some smaller pits that might be induced by the nanoscale inclusion are shown in Figure 11a (marked with red arrows). With time increasing, pits around inclusion enlarged size (Figure 11b). After 30-min immersion test, pits expanded into deeper cavities. This indicated that the pits propagated faster in the vertical direction around the inclusions. After the immersion time increased to 72 h, larger pit holes were formed around inclusion (Figure 11d) and grains in the sample were dissolved profoundly.

Meanwhile, pearlite played a different role in the evolution process of localized corrosion. The typical morphology of pearlites in samples after immersion of 5 min, 30 min and 72 h are shown in Figure 11e–g. After 5 min, the pearlite started to dissolve, but the lamellar Fe_3_C was not obvious. After 30-min immersion, the pearlite was dissolved and the lamellar Fe_3_C was obviously observed on the sample. When the immersion time reached 72 h, the boundary of the lamellar Fe_3_C started to dissolve. During the corrosion developing process, the ferrite in and around the pearlite dissolved without forming deeper pits.

## 4. Discussion

The microstructure of the steel was composed of ferrite and pearlite (Figure 2), Al_2_O_3_ inclusions (2–7 μm) (Figure 1) and nanoscale inclusions (100–500 nm) such as MnS and Al_2_O_3_–MnS (Figure 2).

**Figure 12:**
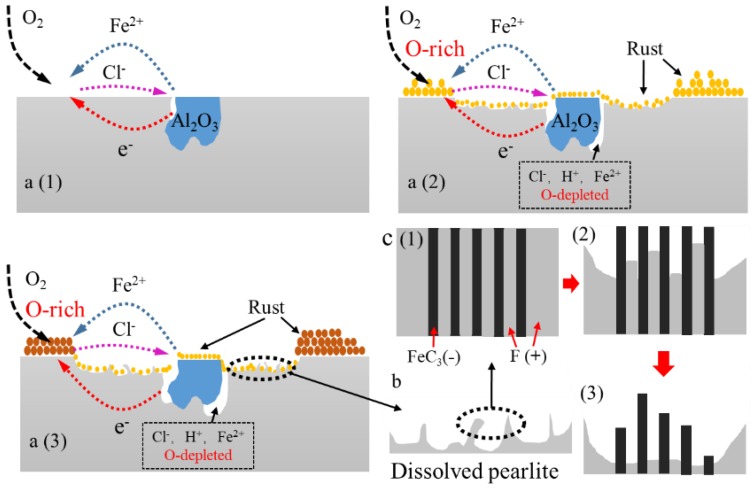
(**a**) Schematic of the localized corrosion initiation and evolution process; and (**b**) diagram of the dissolved pearlite morphology; (**c**) Schematic of the pearlite dissolution process.

With the increasing of immersion time, localized corrosion was formed and evolved on the specimen surface as corrosion spots. According to the tracked corrosion spot propagation process, the mechanism of the localized initiation and evolution process is as shown in Figure 12.

Pits easily formed around the Al_2_O_3_ inclusions. Due to the existence of microcrevices, aggressive ions enriched easily in microcrevices (Figure 12(a_1_)). Crevice corrosion may be formed as a result of the direct exposure of the boundary between the inclusion and the matrix to the aggressive environment [39], which results in localized corrosion (Figure 6b and Figure 10a) [40]. Moreover, the SKPFM result indicates that the matrix with a lower potential was a vulnerable area, which can be easily attacked in the corrosion process [4]. Corrosion products such as Fe^2+^ spread around from the pits, and aggressive ions such as Cl^−^ spread to the pits from further away. Cl^−^ has a tendency to accelerate localized corrosion result from it accumulating inside the catalytic-occluded cells to maintain electroneutrality [41]. This ion migration cycle can maintain the corrosion reaction (Figure 12(a_1_)). The electrons generated in the reaction can transfer through the matrix.

In the localized corrosion initiation and evolution process, corrosion products accumulating on the pit (Figure 6b and Figure 10a) lead to the formation of occluded cells, with the pits acting as anodes (Figure 12(a_2_)) [9,41]. Meanwhile, oxygen concentration cell could be formed due to the difference of oxygen concentration between the occluded area and the rust covered area.

Some nanoscale inclusions, e.g., MnS, Al_2_O_3_–MnS, or Al_2_O_3_, also affect the propagation process of the corrosion spot. The pits formed around nanoscale inclusions were too shallow to maintain an aggressive solution environment inside (Figure 7c). Stewart and Williams [42] indicated that inclusions are the predominant pit nucleation sites, and the lifetime of a metastable pit is directly related to the size of the inclusion particle. Suter et al. [43] also noted that pit nuclei originating from small inclusions (<1 μm) rarely transformed into steadily growing pits [44,45].

In the localized corrosion evolution process, pearlite played an important role. Pearlite was made up of lamellar Fe_3_C and ferrite. The coexistence of cementite and ferrite in pearlite induced a galvanic couple, which was assumed to increase the corrosion of pearlite and to increase the corrosion rate of ferrite–pearlite steel as a whole [37]. Lamellar Fe_3_C in the pearlite can accelerate the dissolution of ferrite as a cathodic phase in the corrosion process (Figure 12(c_1_–c_3_)) [46,47] The lamellar Fe_3_C was clearly observed in the corrosion spot (Figure 7d). Numerous remaining lamellar Fe_3_C (marked with yellow arrows) were observed in the corrosion spot (Figure 7a). The lamellar Fe_3_C could not only accelerate the dissolution of ferrite in pearlite [48], but also accelerate the dissolution of ferrite around the pearlite [49] (Figure 12). Hence, pearlite might be the reason for the localized corrosion evaluation.

In the localized corrosion initiation and evolution process, the cathodic reaction might take place as follows:

1/2O_2_ + H_2_O + 2e^−^ → 2OH^−^ (1)

Fe^2+^ + 2OH^−^ → Fe(OH)_2_ (2)

2Fe(OH)_2_ + 1/2O_2_ + H_2_O + e^−^ → 2Fe(OH)_3_ (3)

In the SVET measurements, the peak value of current density decreased along with the immersion time (Figure 5 and Figure 9). In the 4-h SVET test, the anodic current covering area increased as time increased (Figure 9). As shown in Figure 10b, after immersion for 4 h, the rust covering area was also corroded, which indicated that the corrosion form translated from localized corrosion to uniform corrosion after immersion 4 h.

Cr and Cu may segregate during the dissolution of iron, and may adsorb on the steel surface, thus accelerating the uniform dissolution of the steel [50,51]. Moreover, the presence of Cu retards the growth of rust and suppresses the supply of oxygen to the steel surface; Cu reduces the conductivity of the rust; Cu retards the crystallization of rust and contributes to a uniform dissolution of the steel and the formation of a rust layer at the initial corrosion stage [46]. Hence, the copper and chromium additions can enrich the rust-layer/substrate interface and the rust layers, respectively. They can therefore enhance the compactness and densification of these rust layers [50]. This may result in the decrease of corrosion current, as observed in Figure 5.

In the early stage of the corrosion process, localized corrosion was mainly induced by the Al_2_O_3_ inclusions. Pearlite do not have an important role in this stage. Over time, pits expanded into deeper cavities due to the faster propagation speed in the vertical direction around the inclusion (Figure 12a). The effect of pearlite on the corrosion developing process started to appear as time progressed. The galvanic couple between lamellar Fe_3_C and ferrite could accelerate the dissolution of ferrite in or around the pearlite. Pearlite content resulted in faster dissolution of steel, higher corrosion current density, and in the shift of corrosion potentials to more positive values [47]. The pearlite played an important role in the localized corrosion evolution process. The pearlite accelerated the localized corrosion development in the horizontal direction. Under the synergistic effect of inclusion and the pearlite, the localized rust could continue developing in both the vertical and horizontal directions. However, nanoscale inclusions (smaller than 1 μm) could not induce deep and steady pits [43,44,45] since the small open cavities around nanoscale inclusions could not retain an aggressive environment for corrosion [42].

## 5. Conclusions

In summary, the initiation and evolution of the localized corrosion in carbon steel were investigated in situ in a simulated marine environment of Xisha Island in the South China Sea. The localized corrosion as the form of corrosion spot was initiated with some synergetic effects. The pits that formed around Al_2_O_3_ inclusions due to the dissolution of the matrix was an important factor for the initiation of the corrosion spot. Occluded cells, formed in the pit around the Al_2_O_3_ inclusion with corrosion products covering them, can promote the corrosion process, thereby accelerating the propagation of the corrosion spot. The oxygen concentration cell formed due to the difference of oxygen concentration between occluded area and the rust covered area. This could also accelerate the localized corrosion. Lamellar Fe_3_C in the pearlite could accelerate the dissolution of ferrite as a cathodic phase in the corrosion process due to the galvanic couple effect between Fe_3_C and ferrite. In conclusion, crevice corrosion, occluded cells, oxygen concentration cell and the galvanic couple between Fe_3_C and ferrite can promote the initiation and evolution process of localized corrosion.

## Figures and Tables

**Table 1:** Composition of the nanoscale inclusions in Figure 2 (wt %).

	Fe	Cr	Cu	Si	Nb	C	Mn	S	Al	O
Spectrum 1	83.0	1.9	2.4	0.2	2.4	1.4	5.0	3.7	-	-
Spectrum 2	42.8	0.7	0.8	-	-	0.8	1.3	0.7	25.4	27.3

**Table 2:** Composition of the microstructures in Figure 7 (wt %).

	Fe	Cr	Si	Nb	C	Al	O
Spectrum 1	38.7	-	-	-	-	31.9	29.4
Spectrum 2	33.7	0.7	0.2	0.9	6.6	25.0	32.9
Spectrum 3	74.3	2.4	0.2	-	16.9	-	6.2

## References

[1] Li X., Zhang D., Liu Z., Li Z., Du C., Dong C. (2015). Materials science: Share corrosion data. Nature.

[2] Liu Z.Y., Li X.G., Du C.W., Lu L., Zhang Y.R., Cheng Y.F. (2009). Effect of inclusions on initiation of stress corrosion cracks in X70 pipeline steel in an acidic soil environment. Corros. Sci..

[3] Yang Y., Zhang T., Shao Y., Meng G., Wang F. (2013). New understanding of the effect of hydrostatic pressure on the corrosion of Ni–Cr–Mo–V high strength steel. Corros. Sci..

[4] Liu C., Revilla R.I., Liu Z., Zhang D., Li X., Terryn H. (2017). Effect of inclusions modified by rare earth elements (Ce, La) on localized marine corrosion in Q460NH weathering steel. Corros. Sci..

[5] Williams D.E., Westcott C., Fleischmann M. (1985). Stochastic Models of Pitting Corrosion of Stainless Steels I. Modeling of the Initiation and Growth of Pits at Constant Potential. J. Electrochem. Soc..

[6] Torkkeli J., Saukkonen T., Hänninen H. (2015). Effect of MnS inclusion dissolution on carbon steel stress corrosion cracking in fuel-grade ethanol. Corros. Sci..

[7] Reformatskaya I.I., Rodionova I.G., Beilin Y.A., Nisel’son L.A., Podobaev A.N. (2004). The Effect of Nonmetal Inclusions and Microstructure on Local Corrosion of Carbon and Low-alloyed Steels. Prot. Met..

[8] Jin T.Y., Liu Z.Y., Cheng Y.F. (2010). Effect of non-metallic inclusions on hydrogen-induced cracking of API5L X100 steel. Int. J. Hydrog. Energy.

[9] Ryan M.P., Williams D.E., Chater R.J., Hutton B.M., McPhail D.S. (2002). Why stainless steel corrodes. Nature.

[10] Wei J., Dong J., Ke W., He X. (2015). Influence of Inclusions on Early Corrosion Development of Ultra-low Carbon Bainitic Steel in NaCl Solution. Corrosion.

[11] Melchers R., Chaves I., Jeffrey R. (2016). A Conceptual Model for the Interaction between Carbon Content and Manganese Sulphide Inclusions in the Short-Term Seawater Corrosion of Low Carbon Steel. Metals.

[12] Staicopolus D. (1963). The role of cementite in the acidic corrosion of steel. J. Electrochem. Soc..

[13] Cui N., Qiao L., Luo J., Chiovelli S. (2000). Pitting of carbon steel with banded microstructures in chloride solutions. Br. Corros. J..

[14] Asma R., Yuli P., Mokhtar C. (2011). Study on the effect of surface finish on corrosion of carbon steel in CO_2_ environment. J. Appl. Sci..

[15] Jones D.A. (1996). Principles and Prevention of Corrosion.

[16] Liu C., Revilla R.I., Zhang D., Liu Z., Lutz A., Zhang F., Zhao T., Ma H., Li X., Terryn H. (2018). Role of Al_2_O_3_ inclusions on the localized corrosion of Q460NH weathering steel in marine environment. Corros. Sci..

[17] Bastos A.C., Simões A.M., Ferreira M.G. (2003). Corrosion of Electrogalvanized Steel in 0.1 M NaCl Studied by SVET. Port. Electrochim. Acta.

[18] Isaacs H.S. (1991). The Effect of Height on the Current Distribution Measured with a Vibrating Electrode Probe. J. Electrochem. Soc..

[19] Tang Y., Zuo Y. (2004). The metastable pitting of mild steel in bicarbonate solutions. Mater. Chem. Phys..

[20] Qian H., Xu D., Du C., Zhang D., Li X., Huang L., Deng L., Tu Y., Mol J.M.C., Terryn H.A. (2017). Dual-action smart coatings with a self-healing superhydrophobic surface and anti-corrosion properties. J. Mater. Chem. A.

[21] Zhang D., Qian H., Wang L., Li X. (2016). Comparison of barrier properties for a superhydrophobic epoxy coating under different simulated corrosion environments. Corros. Sci..

[22] Dong C.F., Luo H., Xiao K., Ding Y., Li P.H., Li X.G. (2013). Electrochemical Behavior of 304 Stainless Steel in Marine Atmosphere and Its Simulated Solution. Anal. Lett..

[23] Nishikata A., Zhu Q., Tada E. (2014). Long-term monitoring of atmospheric corrosion at weathering steel bridges by an electrochemical impedance method. Corros. Sci..

[24] Deng Z., Zhu M. (2013). Evolution mechanism of non-metallic inclusions in Al-killed alloyed steel during secondary refining process. ISIJ Int..

[25] Yu H.-L., Liu X.-H., Bi H.-Y., Chen L.-Q. (2009). Deformation behavior of inclusions in stainless steel strips during multi-pass cold rolling. J. Mater. Process. Technol..

[26] Vignal V., Oltra R., Josse C. (2003). Local analysis of the mechanical behaviour of inclusions-containing stainless steels under straining conditions. Scr. Mater..

[27] Szklarska-Smialowska S. (1986). Pitting Corrosion of Metals.

[28] Xue H.B., Cheng Y.F. (2011). Characterization of inclusions of X80 pipeline steel and its correlation with hydrogen-induced cracking. Corros. Sci..

[29] Jeon S.-H., Kim S.-T., Choi M.-S., Kim J.-S., Kim K.-T., Park Y.-S. (2013). Effects of cerium on the compositional variations in and around inclusions and the initiation and propagation of pitting corrosion in hyperduplex stainless steels. Corros. Sci..

[30] Nakhaie D., Moayed M.H. (2014). Pitting corrosion of cold rolled solution treated 17-4 PH stainless steel. Corros. Sci..

[31] Revilla R.I., Liang J., Godet S., Graeve I.D. (2017). Local Corrosion Behavior of Additive Manufactured AlSiMg Alloy Assessed by SEM and SKPFM. J. Electrochem. Soc..

[32] Sathirachinda N., Pettersson R., Wessman S., Pan J. (2010). Study of nobility of chromium nitrides in isothermally aged duplex stainless steels by using SKPFM and SEM/EDS. Corros. Sci..

[33] Jacobs H.O., Knapp H.F., Müller S., Stemmer A. (1997). Surface potential mapping: A qualitative material contrast in SPM. Ultramicroscopy.

[34] Jacobs H.O., Leuchtmann P., Homan O.J., Stemmer A. (1998). Resolution and contrast in Kelvin probe force microscopy. J. Appl. Phys..

[35] Wang L.W., Liu Z.Y., Cui Z.Y., Du C.W., Wang X.H., Li X.G. (2014). In situ corrosion characterization of simulated weld heat affected zone on API X80 pipeline steel. Corros. Sci..

[36] López D.A., Simison S.N., de Sánchez S.R. (2003). The influence of steel microstructure on CO_2_ corrosion. EIS studies on the inhibition efficiency of benzimidazole. Electrochim. Acta.

[37] Xu C., Shi K., Zhou Y., Li X., Liu Y., Wang H. (2011). Microstructures and corrosion properties of X80 pipeline steel in alkaline sand soil. Trans. JWRI.

[38] Keleştemur O., Yıldız S. (2009). Effect of various dual-phase heat treatments on the corrosion behavior of reinforcing steel used in the reinforced concrete structures. Constr. Build. Mater..

[39] Suter T., Böhni H. (2001). Microelectrodes for corrosion studies in microsystems. Electrochim. Acta.

[40] Daud A.R. (1996). Corrosion at sulphide inclusions in stainless steel. Pertanika J. Sci. Technol..

[41] Brossia C.S., Kelly R.G. (1998). Influence of Alloy Sulfur Content and Bulk Electrolyte Composition on Crevice Corrosion Initiation of Austenitic Stainless Steel. Corrosion.

[42] Stewart J., Williams D.E. (1992). The initiation of pitting corrosion on austenitic stainless steel: On the role and importance of sulphide inclusions. Corros. Sci..

[43] Suter T., Böhni H. (1997). A new microelectrochemical method to study pit initiation on stainless steels. Electrochim. Acta.

[44] Khatak H., Raj B. (2012). Corrosion of Austenitic Stainless Steels: Mechanism, Mitigation and Monitoring.

[45] Li N., Wang Y., Qiu S., Xiang L. (2016). Effect of Ce on the Evolution of Recrystallization Texture in a 1.2%Si-0.4%Al Non-oriented Electrical Steel. ISIJ Int..

[46] Hao X., Dong J., Etim I.-I.N., Wei J., Ke W. (2016). Sustained effect of remaining cementite on the corrosion behavior of ferrite-pearlite steel under the simulated bottom plate environment of cargo oil tank. Corros. Sci..

[47] Shibaeva T.V., Laurinavichyute V.K., Tsirlina G.A., Arsenkin A.M., Grigorovich K.V. (2014). The effect of microstructure and non-metallic inclusions on corrosion behavior of low carbon steel in chloride containing solutions. Corros. Sci..

[48] Chen Y.Y., Tzeng H.J., Wei L.I., Wang L.H., Oung J.C., Shih H.C. (2005). Corrosion resistance and mechanical properties of low-alloy steels under atmospheric conditions. Corros. Sci..

[49] Hao L., Zhang S., Dong J., Ke W. (2011). Atmospheric corrosion resistance of MnCuP weathering steel in simulated environments. Corros. Sci..

[50] Haisch T., Mittemeijer E.J., Schultze J.W. (2002). On the influence of microstructure and carbide content of steels on the electrochemical dissolution process in aqueous nacl-electrolytes. Mater. Corros..

[51] Stratmann M., Bohnenkamp K., Ramchandran T. (1987). The influence of copper upon the atmospheric corrosion of iron. Corros. Sci..

